# Bidirectional association between visual impairment and frailty among community-dwelling older adults: a longitudinal study

**DOI:** 10.1186/s12877-022-03365-0

**Published:** 2022-08-15

**Authors:** Tianxue Hou, Minhui Liu, Jinghui Zhang

**Affiliations:** 1 Teaching and Research Section of Clinical Nursing, Xiangya Hospital, Central South University, 87 Xiangya Road, Kaifu District, Changsha, Hunan 410008 People’s Republic of China; 2 Central South University Xiangya School of Nursing, Changsha, Hunan China; 3 Johns Hopkins University School of Nursing, Baltimore, MD USA; 4 National Clinical Research Center for Geriatric Disorders, Xiangya Hospital, Changsha, Hunan People’s Republic of China

**Keywords:** Visual impairment, Frailty, Risk factor

## Abstract

**Background:**

Vision impairment is common among older adults, and it may be related to frailty. However, the longitudinal relationship between visual impairment and frailty is still unclear.

**Methods:**

We used data from Round 1 to Round 5 from the National Health and Aging Trend Study. Two samples were community-dwelling older adults, sample 1 (without visual impairment) was classified according to whether they have pre-frailty/frailty at R1 (*N* = 3013) and sample 2 (without pre-frailty/frailty) was classified according to whether they have visual impairment at R1 (*N* = 1422), respectively. Frailty was measured using five criteria: experiencing exhaustion, unintentional weight loss, low physical activity, slow gait, and weak grip strength. Visual impairment was assessed by asking participants if they had any visual impairment. Generalized estimating equation models were used to examine the concurrent and lagged association between visual impairment and frailty.

**Results:**

The participants were on average 76 ± 7 years old, female (59%), non-Hispanic white (74%) with less than bachelor educated (73%), and 44% were pre-frail/frail in the older adults without visual impairment. Approximately 5% of participants had visual impairment at R1, and they tended to be female and non-Hispanic White in the older adults without frailty. The concurrent (OR, 95% CI = 1.55, 1.17-2.02) and lagged (OR, 95% CI = 1.79, 1.25-2.59) associations between frailty and visual impairment were significantly after controlling the covariates. Similarly, the concurrent (OR, 95% CI = 1.63, 1.32-2.04) and lagged (OR, 95% CI = 1.49, 1.20-1.87) associations between visual impairment and frailty were also significant.

**Conclusions:**

Overall, this study provides evidence for a longitudinal, bidirectional association between self-reported visual impairment and frailty. Future intervention programs to delay frailty progression should include strategies that may reduce the incidence of visual impairment.

## Introduction

Vision impairment is common among older adults. About 3.22 million people in the United States suffer from visual impairment, with the largest percentage (50%) of older adults over 80 years old [1]. Visual impairment negatively affects many aspects of daily functioning, such as physical and cognitive functioning [2–4]. It also associated with the risk of disability, comorbidity, and mortality [5, 6]. This means that visual impairment is a very serious problem, but most moderate to severe visual impairments are correctable (e.g. cataract surgery or refractive error correction) or even preventable (e.g. glaucoma) [7].

Disability is a condition caused by illness or impairment and the interaction of other physical, social, psychological and environmental factors that is difficult to reverse [8]. However, frailty is a reversible condition that precedes disability [9]. Frailty is defined as a clinically recognizable state, increased vulnerability caused by age-related reserves and decreased function of multiple physiological systems [10]. Similarly, older adults with frailty are at risk for adverse health consequences, including falls [11],disability [12], hospitalization [13], and mortality [13]. However, early intervention for older adults with frailty may have a major impact on the health of older adults.

Considering that frailty is important risk factor for disability, and visual impairment is related to poor functional status and disability, thus, research on the relationship between frailty and visual impairment is necessary. At present, relevant research is still very limited. A cross-sectional study with a sample size of 2962 participants over age 43 showed that worse visual acuity and contrast sensitivity were associated with lower frailty scores [14]. However, this cross-sectional study was unable to determine the temporal direction of the association between visual impairment and frailty. In addition, studies have shown that visual impairment is associated with an increased risk of frailty and contributes to the development of frailty [15]. However, no longitudinal study has examined frailty as a risk factor for vision impairment, although there are several hypothetical reasons for such an association. On the one hand, frailty has been associated with behaviors which may increase ocular disease risk, such as low physical activity [16]. On the other hand, older adults with frailty usually take opioids to relieve disease-related pain symptom, which may cause damage to vision [17]. Therefore, vision impairment and frailty may be linked in a two-way fashion, which may produce a downward spiral, where each in turn triggers the other. Understanding how vision impairment and frailty interact over time is important for the health and quality of life of older adults. This includes determining the potential utility of visual impairment screening for older adults at high risk for developing frailty.

We used a nationally representative sample of Medicare beneficiaries from the National Health and Aging Trendy Study (NHATS) to investigate the longitudinal impact of visual impairment on frailty and to investigate the longitudinal impact of frailty on visual impairment. We hypothesized that visual impairment can predicted the onset of frailty, and likewise, frailty may predict the onset of visual impairment.

## Methods

We used data from 2011 Round 1 (R1) to 2015 Round 5 (R5) of the National Health and Aging Trends Study (NHATS), a cohort of nationally representative sample of Medicare beneficiaries aging 65 years and older residing in the United States [18]. The initial NHATS participant cohort was created in 2011 and refreshed in 2015 to replenish participants who dropped out because of death or other reasons [19]. The NHATS was designed to study functioning in later life and includes longitudinal data on both visual impairment and frailty. Two samples were community-dwelling older adults, sample 1 (without visual impairment) was classified according to whether they have pre-frailty/frailty at R1 (*N* = 3013) and sample 2 (without pre-frailty/frailty) was classified according to whether they have visual impairment at R1 (*N* = 1422), respectively. The NHATS was approved by the Johns Hopkins Bloomberg School of Public Health IRB (grant number NIA U01AG032947) [18]. NHATS participants completed written informed consent prior to being interviewed.

### Measures

#### Frailty

Frailty was measured using the physical frailty phenotype determined by five criteria: exhaustion, low physical activity, weakness, slowness, and shrinking [20]. If participants reported they had low energy or being easily exhausted (enough to limit their activities) recently, they met the first criteria [21]. If participants reported they never walked for exercise or engaged in vigorous activities recently, they met the second criteria [21]. If participants reported they had body mass index (BMI) less than 18.5 kg/m^2^, based on self-reported height and weight, or reported unintentionally losing 10 or more pounds in the last year, they met the third criteria [21]. If the result of using the first of two usual pace walking trials was at or below the 20th percentile of the weighted population distribution within four sex-by-height categories, they met the fourth criteria [20]. If the result of using maximum dominant hand grip strength over two trials, as being at or below the 20th percentile within eight sex-by-BMI categories, they met the fifth criteria. Participants who met three or more criteria were considered as “frailty” [20]. Those with one or two criteria were considered as “pre-frailty,” and those without any criterion as “robust.” [21].

#### Visual impairment

Visual impairment (VI) was assessed using responses to self-reported questionnaires [19]. Participants were classified as having any VI (impairment at distance or near, distance impairment specifically, inability to “recognize someone across the street” or “watch television across the room”, and inability to “read newspaper print”, even with glasses), and no VI [19].

### Covariates

#### Sociodemographic

Basic demographic information including age, sex (e.g. male, female), race (e.g. non-Hispanic White,), education (e.g. less than high school, high school graduates), living arrangement (e.g. alone, with spouse only) were recorded.

#### Health-related and behavioral factors

Health-related factors included body mass index (BMI) (normal, underweight which is defined as a BMI less than 18.5 kg/m^2^, and overweight which is defined as a BMI greater than or equal to 25 kg/m^2^); the number of chronic illnesses (i.e., high blood pressure, heart attack/heart disease, arthritis, osteoporosis, diabetes, lung disease, stroke, and cancer) diagnosed by a doctor (none, 1-3 diseases, or more than 4 diseases); whether or not the participants were hospitalized in the last 12 months (no hospitalization, at least one hospitalization); self-rated health (excellent, very good, good, fair, or poor); cognitive function (no dementia; possible dementia; probable dementia); and smoking (never smokers, current/former smokers).

#### Statistical analyses

Descriptive statistics were presented as mean ± SD (standard deviation) for continuous variables or absolute number and percentage for categorical variables. Chi-square tests were used to compare the baseline characteristics of the sample according to frailty categories (robust, pre-frail/ frail) and visual impairment (yes or no).

The effect of baseline frailty on the concurrent and lagged incident vision impairment or of baseline vision impairment on the concurrent and lagged incident frailty was analyzed using generalized estimating equation (GEE) models to estimate Odds Ratio (OR) and 95% confidence intervals (CIs) for the association between visual impairment and frailty, which specify the logit link function with a binomial distribution and assume an exchangeable correlation structure and used robust standard errors to account for the correlation between measures for each measure. Briefly, GEE model has two basic characteristics: 1) accounts for the within-subject correlation across repeated measurements. 2) is appropriate to estimate population-averaged effects over time [22]. The statistics of missing data are ranged from 0.2% (Smoking variable) to 8% (BMI variable). Given the small percentage of missing data and large sample size, we did not use techniques to handle missing data.

We used four sets of GEE models. In the first set of models, we assessed the concurrent association between vision impairment and frailty with adjustment for the visual impairment in the prior year. In the second set of models, we used the visual impairment status at the baseline to predict one-year-lagged frailty adjustment for concurrent visual impairment. In the third set of models, we assessed the concurrent association between frailty and vision impairment with adjustment for the frailty in the prior year. In the fourth set of models, we used the frailty status at the baseline to predict one-year-lagged visual impairment with adjustment for concurrent frailty.

In the first two sets of models, we excluded the participants who had been having visual impairment at the baseline. The first set of models consists of three models altogether. In Model 1, we presented the crude associations initially. In Model 2, odds ratios (OR) were adjusted for demographics (chronological age, gender, race, education, and living arrangement). In addition, Model 3 based on the model 2 included health-related covariates (pain, depression, BMI, health status, number of chronic illnesses, hospitalization, and smoking). In the last two sets of models, we excluded the participants who had been having frailty at the baseline. The last two sets of models controlled covariates were the same as those controlled covariates by the first set of models.

We also performed a sensitivity analysis. We restricted the sample to participants 65 to 85 years of age to reduce possible residual confusion due to age. All statistical analyses were performed using Stata version 15. 0 (Stata Corp, College Station, Texas, USA); *p* < .05 (two-tailed) was used to indicate statistical significance.

## Results

### Sample characteristics

In the first sample (non-visual impairment), the average ages were 75.6 ± 6.9 years old, 59% were female, 74% were non-Hispanic whites, 72% were less than bachelor educated, 46% lived with spouse/partner only, and 9% had possible dementia (*N* = 3013). Baseline frailty assessments grouped participants as robust (*n* = 1327, 44%) and pre-frail/frail (*n* = 1686, 56%). The older adults with frailty were more likely to have good self-rated health status, no dementia, and more chronic illnesses. (Table 1).

**Table 1:** Demographic and Health Comorbidity Characteristics of the NHATS Study Populations at Round 1

Variables	Total	Pre-frail/frail (*N* = 3013)			Total	Visual Impairment (*N* = 1422)		
		No (*n* = 1327)	Yes (*n* = 1686)	*P* value		No (*n* = 1358)	Yes (*n* = 64)	*P* value
Age								
Years, mean (SD)	75.6 ± 6.9	74.5 ± 6.6	76.5 ± 7.1		74.7 ± 6.7	74.6 ± 6.6	77.1 ± 7.9	
Gender				**<0.001**				0.2
Female	58.7	54.2	62.2		54.8	54.3	62.5	
Male	41.3	45.8	37.8		45.2	45.7	37.5	
Race/Ethnicity				**<0.001**				**0.026**
Non-Hispanic White	74	78.8	71.1		77.3	77.6	70.3	
Non-Hispanic Black	19.3	15.9	22.2		15.7	15.8	15.6	
Indian/Asian/Native/Hawaii	2	2	1.8		2.3	2.2	3.1	
Hispanic	4	3.3	4.6		3.7	3.3	10.9	
Other	0.7	0	0.4		1.1	1.1	0	
Education				**<0.001**				**0.038**
Less than high school	19.8	13.4	24.6		14.1	13.9	20.3	
High school graduates	26.7	23.5	29.2		23.9	23.3	34.4	
Some college or vocational school	25.9	25.6	26.2		25.4	25.6	21.9	
Bachelor or higher	27.6	37.5	20		36.6	37.2	23.4	
Living arrangement				**<0.001**				0.238
Alone	32.8	31.5	34.7		31.6	31.3	39.1	
With spouse/partner only	45.5	51.9	38.8		51.5	52.1	39.1	
With others only	12.9	9.5	16.1		9.7	9.6	12.5	
With spouse/partner and with others	8.9	7.1	10.4		7.1	7	9.4	
Self-rated health				**<0.001**				**0.017**
Excellent	15.7	24.6	8.7		24.1	24.5	18.8	
Very good	32.3	40.4	26		39.7	40.1	32.8	
Good	32.2	28.1	35.7		28.4	27.9	37.5	
Fair	15.6	6.2	22.8		6.7	6.7	6.3	
Poor	4.2	0.8	6.9		1.1	0.9	4.7	
BMI				**<0.001**				**0.017**
Normal	28.9	32.5	26.2		32.1	32.2	29.7	
Underweight	1.4	0	2.4		1.1	0.9	4.7	
Overweight	69.7	67.5	71.4		66.9	66.9	66.9	
Hospitalized				**<0.001**				0.173
No	81.2	88.8	75.4		88.2	88.4	82.8	
Yes	18.8	11.2	24.6		11.8	11.6	17.2	
Number of chronic illnesses				**<0.001**				0.131
No disease	9.4	13.5	6.1		13.2	13.4	9.4	
1-3	69.2	74.3	65		74.2	74.4	70.3	
≥ 4	21.5	12.1	28.9		12.6	12.2	20.3	
Smoking				0.799				0.794
No	49.4	50	48.9		50	50.1	48.4	
Yes	50.6	50	51.1		50	48.9	51.6	
Pain				**<0.001**				0.453
No	46	59.5	35.2		59.5	59.4	64.1	
Yes	54.1	40.5	64.8		40.5	40.7	35.9	
Depression				**<0.001**				0.108
No	89.1	95.2	84.3		94.9	95.1	90.5	
Yes	10.9	4.8	15.7		5.1	5	9.5	
Cognitive Function				**<0.001**				**0.018**
No dementia	87.7	91.9	84.4		90.9	1.3	81.3	
Possible dementia	9.4	6.5	11.6		7.4	7.1	14.1	
Probable dementia	2.9	1.6	4.0		1.8	1.6	4.7	

*Note*. The statistics used Pearson χ2

The cell values were vertical percentages

In the second sample (non-frailty), the average ages were 74.7 ± 6.7 years old, 54% were female, 78% were non-Hispanic whites, 63% were less than bachelor educated, 52% lived with spouse/partner only, and 7% had possible dementia (*N* = 1422). Baseline visual impairment assessments grouped participants as no visual impairment (*n* = 1358, 95%), visual impairment (*n* = 64,5%). The participants with visual impairment were more likely to have less than high school educational level, have good self-rated health status, no dementia, and be smokers. (Table 1).

### Visual impairment as predictors of pre-frailty or frailty

**Fig. 1:**
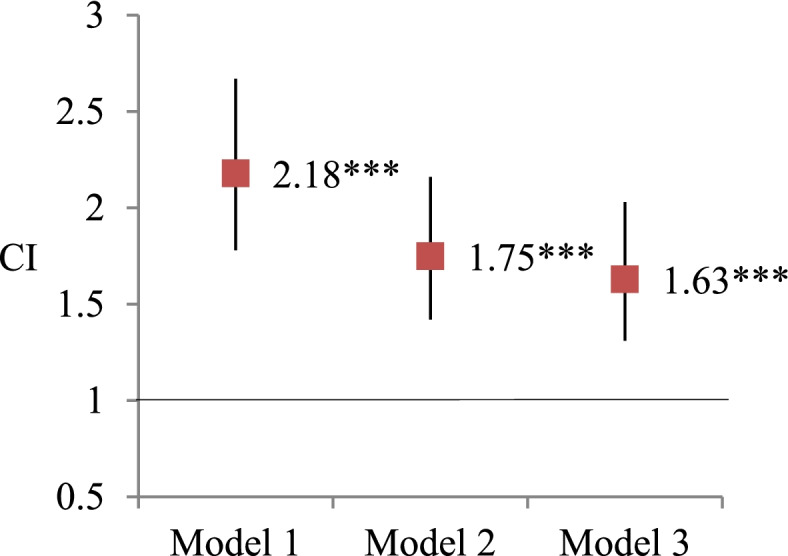
Analysis of the concurrent association of visual impairment predicting frailty by generalized estimating equations.  Note. * *P* < 0.05, ** *P* < 0.01, ***P < 0.001. Odds ratio and 95% confidence interval (CI) were reported here. Model 1: independent variables of interest. Model 2: Model 1+ demographic covariates (age, sex, education, race/ethnicity, living arrangement) Model 3: Model 2 + health related covariates (smoking, BMI, vigorous activity, number of chronic illnesses, hospitalization, pain, depression)

**Fig. 2:**
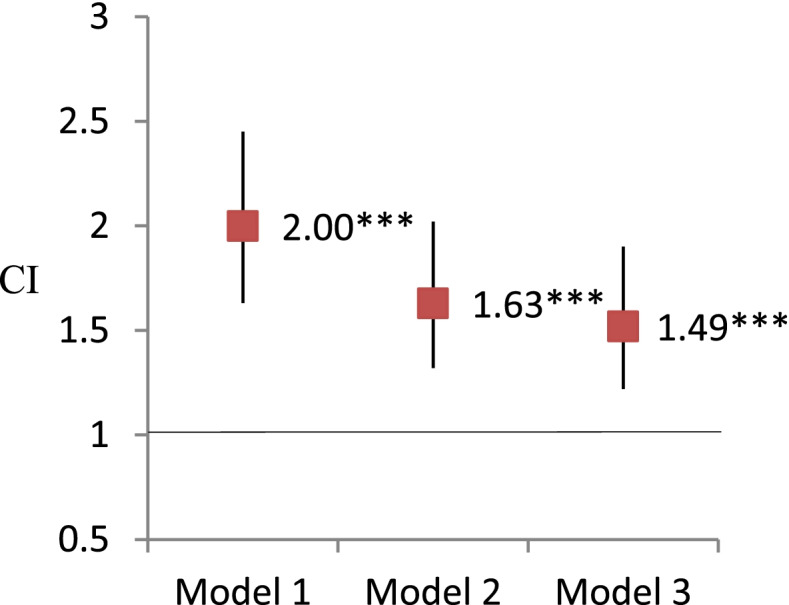
Analysis of the lagged association of frailty predicting visual impairment by generalized estimating equations. Note. * *P* < 0.05, ** *P* < 0.01, ***P < 0.001. Odds ratio and 95% confidence interval were reported here. Model 1: independent variables of interest. Model 2: Model 1+ demographic covariates (age, sex, education, race/ethnicity, living arrangement) Model 3: Model 2 + health related covariates (smoking, BMI, vigorous activity, number of chronic illnesses, hospitalization, pain, depression)

Figures 1 and 2 show the results of GEE models using visual impairment to predict frailty. The concurrent association between visual impairment and frailty was significant in the models after adjusting demographic and health-related variables (OR, 95% CI = 1.63, 1.32-2.04). Same as the concurrent associations, the 5-year lagged associations between visual impairment and frailty were also significant in all adjusted models (OR, 95% CI = 1.49, 1.20-1.97).

### Pre-frailty or frailty as predictors of visual impairment

**Fig. 3:**
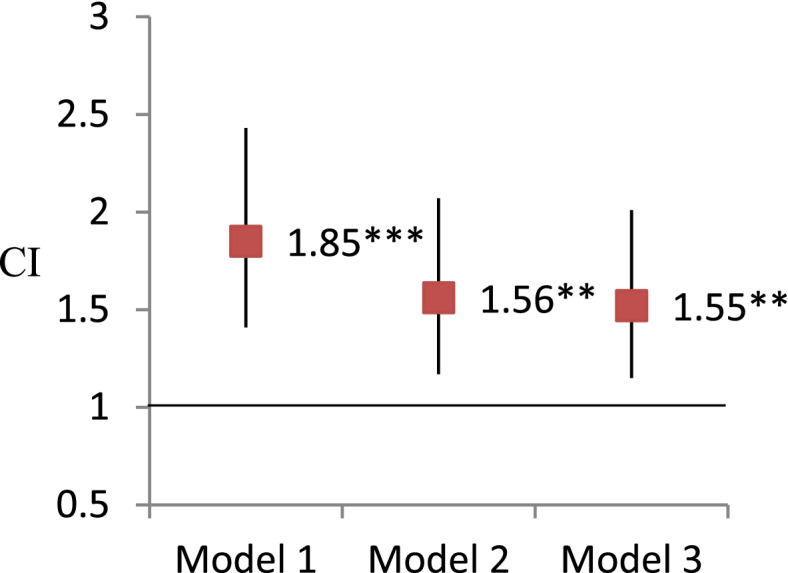
Analysis of the concurrent association of frailty predicting visual impairment by generalized estimating equations. Note. * *P* < 0.05, ** *P* < 0.01, ***P < 0.001. Odds ratio and 95% confidence interval were reported here. Model 1: independent variables of interest. Model 2: Model 1+ demographic covariates (age, sex, education, race/ethnicity, living arrangement) Model 3: Model 2 + health related covariates (smoking, BMI, vigorous activity, number of chronic illnesses, hospitalization, pain, depression)

**Fig. 4:**
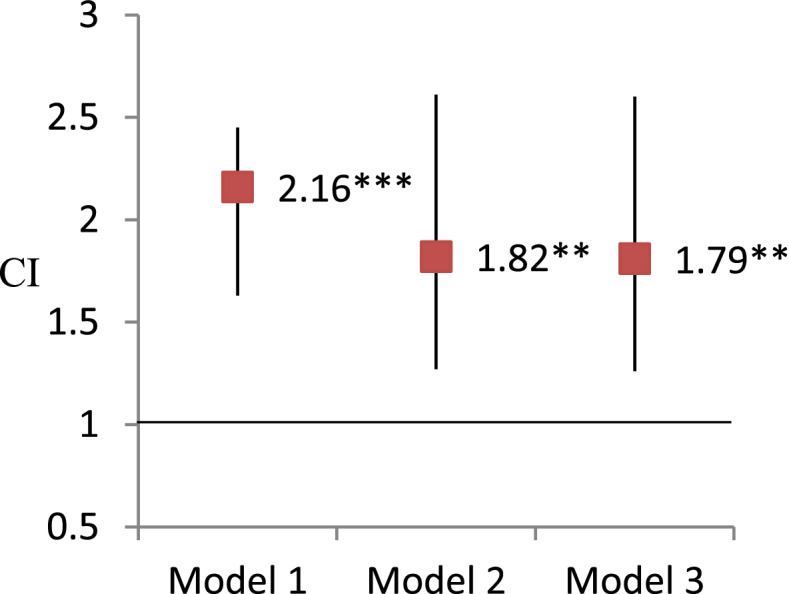
Analysis of the lagged association of frailty predicting visual impairment by generalized estimating equations. Note. * *P* < 0.05, ** *P* < 0.01, ***P < 0.001. Odds ratio and 95% confidence interval (CI) were reported here. Model 1: independent variables of interest. Model 2: Model 1+ demographic covariates (age, sex, education, race/ethnicity, living arrangement) Model 3: Model 2 + health related covariates (smoking, BMI, vigorous activity, number of chronic illnesses, hospitalization, pain, depression)

Figures 3 and 4 present the results of GEE analysis using frailty status to predict visual impairment. The concurrent association between frailty and visual impairment was significant, even after adjusting demographic and health-related variables (OR, 95% CI = 1.55, 1.17-2.06). The 5-year lagged associations between frailty and visual impairment were still significant in the adjusted models (OR, 95% CI = 1.79, 1.25-2.59).

According to the estimates of the sensitivity analysis, for participants between 65 and 85 years old, the results of the study are consistent with the main analysis direction. After controlling for covariates, frailty can predict the occurrence of visual impairment, and vice versa.

## Discussion

To our knowledge, this study was the first attempt to examine the bidirectional association between visual impairment and frailty. Using a nationally representative sample of Medicare beneficiaries from the NHATS, our study demonstrates a strong temporal association between visual impairment and frailty over 5 years of follow-up. When adjusted all the covariates, including sociodemographic, health-related and behavioral factors, we found that visual impairment independently predicted frailty, and frailty also independently predicted visual impairment.

Consistent with the limited literature, visual impairment was associated with increased frailty. A longitudinal study of a sample population of older adults has shown that older adults with visual impairment are more likely to have frailty than those without visual impairment demonstrating a temporal correlation between visual impairment and frailty [10]. Other study has shown that visual impairment in old age can contribute to debilitating development [15]. Compared with older adults without frailty and visual impairment, participants who without frailty, but with visual impairment had a two-fold increased risk of becoming frail before or after follow-up, and this association remained after further adjustment for covariates [15]. On this basis, our study demonstrates a bidirectional predictive relationship between visual impairment and frailty. Using data from a nationally representative study sample (NHATS), it is possible to generalize our results to a broader group of older adults.

The relationship between frailty and visual impairment may be related to the following mechanisms. Oxidative stress, chronic inflammation, and mitochondrial dysfunction play important roles in age-related muscle wasting, including sarcopenia, which is an important cause of frailty [23]. On the one hand, older adults with visual impairments have higher levels of oxidative stress, and some visual impairments share antecedents with chronic inflammation [24, 25]. On the other hand, mitochondrial dysfunction and mitochondrial diseases are associated with the occurrence of visual impairment [26]. Importantly, the more severe the visual impairment, the greater the risk of developing sarcopenia and the higher risk of frailty [27].

.Over time, the effects of visual impairment on frailty may be amplified by the effects of frailty on visual impairment. To our knowledge, no population-based longitudinal study has previously investigated the bidirectional relationship. After adjusting for a large number of possible confounders, including sociodemographic factors (age, sex, education, etc.), disease (hypertension, diabetes, etc.), and behavioral factors (obesity), we found frailty was still a significant predictor of visual impairment. Several factors could explain this relationship. First, a series of comorbidities related to frailty are known to be associated with visual impairment, such as cardiovascular disease and diabetes [28]. Second, common pathological pathways, such as inflammation, have been associated with visual impairment and frailty [29, 30]. Third, due to the lack of exercise for older adults with frailty, it is easy to lead them to social isolation, which has been shown to be related to visual impairment [10, 31]. These factors may lead to a vicious circle.

Our findings have implications for clinical practice. Health care providers may consider referring older adults with frailty and with visual impairment to an ophthalmologist for treatment. Intervention through medication or surgery can improve treatable visual impairment, which may reduce the risk of further frailty. On the contrary, when evaluating visually impaired patients, ophthalmologists may recommend simultaneous frailty screening for those with poor eyesight. Our findings provide important information for the causal relationship between visual impairment and frailty, as well as frailty and ophthalmic interventions, to benefit patients most vulnerable to these injuries, and to help prospectively determine screening guidelines.

The strength of this study includes using NHATS, a nationally representative longitudinal cohort study, which provides us with a large sample size and comprehensive covariate related questions [32]. We acknowledge that our study has limitations. Visual impairment was measured by subjective questions, which can lead to recall or other biases. In addition, due to resource constraints, no formal vision assessment and objective measurement was conducted, and no specific cause of vision loss was identified [19]. However, there are studies that have found significant associations between objectively measured visual impairment and frailty, which supports our findings about the association between visual impairment and frailty [6]. Second, we only used physical frailty phenotype to measure frailty in this study, but according to previous literature, different frailty measures are based on different frailty conceptions and theory models, so there was some heterogeneity in frailty prevalence and associated factors according to different measures, which may affect the correlation between frailty and visual impairment. Different frailty scales could be used in future studies to measure frailty and verify the association between frailty and visual impairment.

## Conclusion

Overall, this study provides evidence for a longitudinal, bidirectional association between self-reported visual impairment and frailty. Given that visual impairment and frailty are two commonly treatable and interrelated conditions. Therefore, timely screening of older adults for visual impairment and frailty may help alleviate the development of disease and disability, and improve the quality of life of older adults.

## Data Availability

The NHATS data analyzed in the current study are available for research purposes at www.nhats.org.

## Abbreviations

VI: Visual Impairment
NHATS: National Health and Aging Trends Study
GEE: Generalized Estimating Equations
IRR: Incidence rate ratio
BMI: Body Mass Index
CI: Confidence Interval
